# Role of Lung P450 Oxidoreductase in Paraquat-Induced Collagen Deposition in the Lung

**DOI:** 10.3390/antiox11020219

**Published:** 2022-01-24

**Authors:** Nataliia Kovalchuk, Joseph L. Jilek, Laura S. Van Winkle, Nathan J. Cherrington, Xinxin Ding

**Affiliations:** 1 Department of Pharmacology and Toxicology, College of Pharmacy, University of Arizona, Tucson, AZ 85721, USA; kovalchuk.nataliia@gmail.com (N.K.); jljilek@pharmacy.arizona.edu (J.L.J.); cherring@pharmacy.arizona.edu (N.J.C.); 2 Department of Anatomy, Physiology and Cell Biology, Center for Comparative Respiratory Biology and Medicine, School of Veterinary Medicine and Center for Health and the Environment, University of California at Davis, Davis, CA 95616, USA; lsvanwinkle@ucdavis.edu

**Keywords:** paraquat, cytochrome P450, P450 reductase, lung fibrosis, airway toxicity

## Abstract

Paraquat (PQ) is an agrochemical known to cause pulmonary fibrosis. PQ-induced collagen deposition in the lung is thought to require enzymatic formation of PQ radicals, but the specific enzymes responsible for this bioactivation event in vivo have not been identified. We tested the hypothesis that lung P450 oxidoreductase (POR or CPR) is important in PQ-induced lung fibrosis in mice. A lung-*Cpr*-null mouse model was utilized, which undergoes doxycycline-induced, Cre recombinase-mediated deletion of the *Por* gene specifically in airway Club cells and alveolar type 2 cells in the lung. The lungs of lung-*Cpr*-null mice and their wild-type littermates were collected on day 15 after a single intraperitoneal injection of saline (control) or PQ (20 mg/kg). Lung tissue sections were stained with picrosirius red for detection of collagen fibrils. Fibrotic lung areas were found to be significantly smaller (1.6-fold for males and 1.4-fold for females) in PQ-treated lung-*Cpr*-null mice than in sex- and treatment-matched wild-type mice. The levels of collagen in lung tissue homogenate were also lower (1.4–2.3-fold; *p* < 0.05) in PQ-treated lung-*Cpr*-null mice compared to PQ-treated wild-type mice. In contrast, plasma PQ toxicokinetic profiles were not different between sex-matched wild-type and lung-*Cpr*-null mice. Taken together, these results indicate that lung POR plays an important role in PQ-induced pulmonary fibrosis.

## 1. Introduction

Paraquat (PQ; 1,1′-dimethyl-4,4′-bipyridylium dichloride) is an effective, fast-acting, and non-selective herbicide with a 50-year history of application and research. This chemical, which has been banned for agricultural use in the European Union due to toxicity, is a restricted-use pesticide in the USA and may be used by certified pesticide applicators to control weeds and grasses in agricultural and non-agricultural areas [1,2]. It is widely used in developing countries as an inexpensive and effective non-selective herbicide [3,4,5,6]. Inhalation and dermal PQ exposures are prevalent in occupational settings.

One of the adverse effects of PQ in mammals, including humans, is a severe acute toxicity that predominantly affects the respiratory system, for which there is no specific antidote. There is a good agreement in the literature that the initial step in PQ-induced damage to airway epithelia is the formation of the PQ radical by cellular diaphorases, which encompass a number of NADH or NADPH dehydrogenases or oxidoreductases [7,8]. The PQ radical undergoes redox cycling, e.g., in airway Club cells and alveolar type II (AT2) epithelial cells, generating ROS (reactive oxygen species), which may initiate PQ-induced cellular oxidative injury through lipid peroxidation, NADPH oxidation, and mitochondrial toxicity [7,9,10,11,12]. These changes trigger an antioxidant response, as illustrated by the induction of xCT (main subunit of cystine/glutamate transporter) and GCL (glutamate cysteine ligase, the rate-limiting enzyme of GSH synthesis) after a single IP injection of 45 mg/kg PQ in mice [13]. Similarly, induction of glutamate cysteine ligase, as well as thioredoxin 1, IL6, IL13, and Stat3, in the lungs of C57BL/6J mice was observed six hours after an intranasal instillation of different doses of PQ, as markers of acute antioxidant and inflammatory responses that preceded lung fibrosis [14]. The extent of deposition of collagen I and fibronectin, two major fibrotic proteins of the extracellular matrix, was elevated in the lungs from C57BL/6J mice exposed to PQ by pharyngeal aspiration [15].

The potential of several mammalian NADPH-dependent diaphorases, localized in different cellular compartments, to mediate PQ redox cycling has been demonstrated. These include activities of cytochrome P450 reductase (POR or CPR) and diphenylene iodonium (DPI)-sensitive thioredoxin reductase in a lysate of MLE-15 murine lung epithelial cells [16], POR in a lysate of Jurkat cell line derived from human peripheral blood T-lymphocytes [17], and the oxidoreductase in the electron transport Complex I in yeast mitochondria [18]. In addition, ATP7A (copper transporter) and SLC45A4 (sucrose transporter) have been identified as essential for PQ-induced cell death [17]. However, the specific enzymes responsible for PQ bioactivation in vivo have not been identified, which hinders progress to identify individuals predisposed to PQ toxicity or to design therapeutic interventions.

In this study, we investigated the contribution of POR of airway epithelial cells to PQ-induced lung fibrosis, utilizing a lung-*Cpr*-null mouse model. This tissue-specific knockout mouse model has been employed previously to demonstrate the specific roles of lung POR-dependent microsomal cytochrome P450 (CYP) enzymes in the acute lung toxicity induced by a ubiquitous pollutant, naphthalene, which causes airway epithelial cell death, but does not induce lung fibrosis, and the lung tumorigenesis induced by a tobacco-specific carcinogen, 4-(methylnitrosamino)-1-(3-pyridyl)-1-butanone [19,20]. In the lung-*Cpr*-null mouse, the *Cpr* (*Por*) gene undergoes doxycycline (DOX)-induced, Cre recombinase-mediated deletion specifically in airway Club cells and AT2 cells, where POR is enriched, in the lung. Lung-*Cpr*-null mice and their WT control littermates, both male and female, were compared on C57BL/6 genetic background for extents of PQ-induced lung fibrosis (sizes of lung areas with collagen fibril deposition detected on picrosirius red-stained tissue sections and levels of mature collagens in lung tissue homogenate). Plasma PQ levels were also determined to confirm the absence of any notable differences between the WT and null mice in systemic PQ metabolism. Our findings demonstrate, for the first time, that lung POR plays an important role in PQ-induced pulmonary fibrosis in vivo.

## 2. Materials and Methods

### 2.1. Chemicals and Reagents

PQ (1,1′-dimethyl-4,4′-bipyridinium dichloride, CAS No. 75365-73-0), ammonium formate, collagen I, picric acid, Sirius red (Direct Red 80) were purchased from Sigma–Aldrich (St. Louis, MO, USA). Acetonitrile and water were of HPLC grade or better (Fisher Scientific, Houston, TX, USA).

### 2.2. Animal Experiments

**Figure 1:**
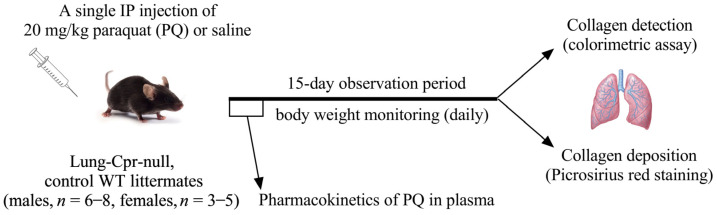
Experimental design to evaluate paraquat (PQ)-induced collagen deposition in the mouse lung. PQ or saline was administered as a single intraperitoneal injection to lung-*Cpr*-null and WT control littermates, male and female. Animals were sacrificed on day 15 after the injection, for harvesting of the lungs for colorimetric analysis and histopathological determinations of collagen deposition. A separate set of lung-*Cpr*-null and WT control littermate mice were used for determination of PQ in plasma following a single intraperitoneal injection of 20 mg/kg PQ.

All procedures were approved by the University of Arizona Institutional Animal Care and Use Committee, Tucson, AZ, USA. Lung-*Cpr*-null (CCSP-rtTA^+/−^/tetO-Cre^+/−^/Cpr^lox/lox^) and “wild-type” control littermates (CCSP-rtTA^+/−^/Cpr^lox/lox^ or tetO-Cre^+/−^/Cpr^lox/lox^), on C57BL/6 genetic background, were generated as described recently [20]. Mice were maintained in a light and temperature-controlled room (12/12-h light-dark cycle, 20 ± 2 °C), with free access to food and water. Adult (3–5-month-old) males and females were used for all experiments. The experimental design is outlined in Figure 1. Mice were maintained on DOX-containing chow diet (625 mg/kg; Envigo Teklad Diets, Madison, WI, USA; catalog No. TD.02503), until 3–4 days before treatment with PQ, when they were changed to a regular (DOX-free) chow (Envigo 7013). Pulmonary fibrosis was induced by a single intraperitoneal injection of 20 mg/kg PQ. PQ was prepared in sterile 0.9% saline [21]. Age-, sex- and genotype-matched control mice received an intraperitoneal injection of sterile saline. Body weight of the treated mice was monitored daily. Mice were euthanized by CO_2_ inhalation and tissues were harvested on day 15 after PQ injection. The left lung was fixed in a large volume of 10% neutral buffered formalin and then embedded in paraffin. The right lung was stored at −80 °C until further analysis.

For toxicokinetic studies, blood samples were collected from PQ-treated males (*n* = 4) and females (*n* = 3) via the tail vein using heparin-coated capillary tubes, at 0.25, 0.5, 1, 2, 4, and 8 h following PQ injection (at 20 mg/kg, i.p.). Plasma, prepared by centrifugation of blood samples at 8000 rpm in an Eppendorf 5424 R centrifuge for 8 min at 4 °C, was stored in sealed tubes at −80 °C until analysis.

### 2.3. PQ Detection

A stock solution of 10 mg/mL PQ was prepared by dissolving PQ powder in deionized water. Working solutions (10–1000 µg/mL) of PQ standards were prepared by diluting the stock solution in an acetonitrile:water (80:20) mixture. Fifteen microliters of plasma samples were diluted in 15 µL of deionized water and 120 µL of acetonitrile was added. The mixture was vortexed for 15 min at room temperature and centrifuged at 12,000 rpm for 10 min in an Eppendorf 5424 R centrifuge. The supernatant was transferred to a new vial and 5 µL of the sample was injected for HPLC-UV analysis.

The HPLC-UV system included an Agilent model 1290 Infinity Series LC and Agilent Infinity model 1260 DAD detector (Agilent Technologies, Santa Clara, CA, USA). HPLC conditions for PQ detection was adopted from published protocols [22,23,24]. Chromatographic separation was performed at room temperature on a XBridge amide column (3.5 µm, 2.1 × 100 mm) connected to a XBridge BEH amide VanGuard cartridge (3.5 µm, 2.1 × 5 mm) (Waters, Milford, MA, USA). The mobile phase was a mixture of solvent A (200 mM ammonium formate in water) and solvent B (acetonitrile) delivered at a flow rate of 0.25 mL/min. The following mobile phase gradient was used: 20%A 0–1 min; linear increase from 20% to 80%A, 1–5 min; hold at 80%A, 5–6 min; linear decrease from 80% to 20%A, 6–7 min; and hold at 20%A, 7–14 min. Analytes were detected by monitoring UV absorbance at 257 nm [22]. Samples for six-point calibration curves, which were constructed by adding authentic PQ standards (0.1–5.0 µg/mL) to the plasma from naïve mice, were processed along with plasma samples from PQ-treated mice. The lower limit of quantification (signal to noise ratio >10) for PQ was 0.1 µg/mL, the retention time for PQ was ~4.7 min, and the analyte recovery from the plasma was ~95%.

### 2.4. Quantification of Lung Fibrosis with Polarized Light Microscopy

Lung sections (5-µm thickness) of left lung from PQ-treated and control mice were stained with picrosirius red following standard protocols [15,25] to evaluate the deposition of total collagen. The slides were deparaffinized, rehydrated and incubated with Weigert’s hematoxylin for 8 min. Washed slides were then immersed in a 0.1% Sirius red solution in aqueous saturated picric acid for 1 h, followed by washing in water, dehydration, and mounting. Stained lung sections were analyzed using a Leica DMI6000B microscope (Leica Microsystems, Buffalo Grove, IL, USA) fitted with filters to provide polarized illumination. Images were observed at ×200 magnification and recorded under brightfield or polarized light under the same conditions (exposure time) with a DFC450 color digital camera that is linked to a computer running the LAS X version 3.3 software (Leica Microsystems, Buffalo Grove, IL, USA). Images captured under brightfield light were used for structural identification, whereas those taken under polarized light were used for quantification.

Images (7–12 per lung section) were analyzed with use of NIH ImageJ (https://imagej.nih.gov/ij/; 30 October 2021) [26]. Blood vessels were manually excluded from quantification as they show similar levels of collagen deposition in the control and PQ-treated groups. Manual exclusion was performed using the freehand selection button in ImageJ software. Threshold value for collagen detection was kept constant for all images analyzed. The area occupied by collagen deposit was calculated as a proportion of the total area of an image analyzed, and expressed in percentage (%). The average percentage of all images for each mouse was calculated and used to obtain the means ± S.D. for each group (*n* = 5–8 for males; *n* = 3–4 for females).

### 2.5. Spectrophotometric Detection of Collagen in Lung Homogenate

Total collagen was detected spectrophotometrically using the picrosirius red assay [27,28]. The lungs were homogenized in CHAPS detergent buffer (50 mM Tris-HCl pH 7.4, 150 mM NaCl, 10 mM CHAPS), with a tissue weight to buffer volume ratio of 1:4. Ten µL of the lung homogenate and 140 µL of 1X PBS were added to each well of a 96-well microtiter plate in duplicate. The plate was dried overnight at 37 °C and subsequently washed three times with distilled water (200 µL/well, 2–3 min each time). To each well, 150 µL of 0.1% Sirius red stain in saturated picric acid was added and the plate was incubated for 1 h at room temperature with rocking (~80 strokes per minute). The plate was then washed four times with acidified water (5% acetic acid, 200 µL/well; 2–3 min each time) and incubated with 150 µL of 0.1 M NaOH for 30 min at room temperature with rocking. The content of each well was transferred to a new 96-well plate, and analyzed for absorbance at 550 nm. Collagen I was used as a standard to prepare calibration curves (0–200 µg/mL in a total well volume of 150 µL).

### 2.6. Statistical Analysis

Pharmacokinetic parameters were calculated using the WinNonlin software (Pharsight, Mountain View, CA, USA). One-way ANOVA with Bonferroni post-hoc multiple comparisons test was used to analyze the differences among groups. The difference was considered to be significant when *p* < 0.05. All analyses were carried out using Prizm version 8 (GraphPad Software, San Diego, CA, USA).

## 3. Results

### 3.1. Pharmacokinetics of Plasma PQ in WT and Lung-Cpr-Null Mice

**Figure 2:**
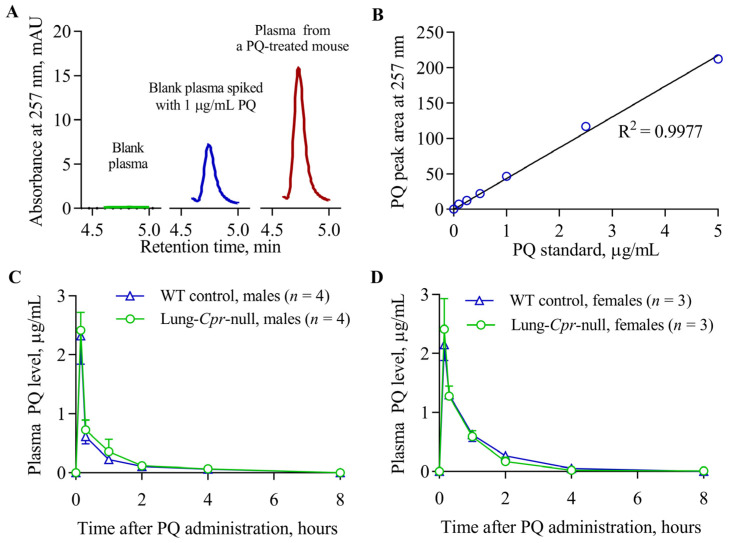
Levels of PQ in plasma following intraperitoneal injection. Three- to four-month-old lung-*Cpr*-null males and females and their age- and sex-matched WT control littermates received a single intraperitoneal dose of PQ at 20 mg/kg. (**A**) Representative chromatograms of PQ in plasma: (**green**) a blank sample; (**blue**) a blank mouse plasma sample supplemented with PQ standard (1 µg/mL); and (**red**) a mouse plasma sample obtained at 15 min after a single intraperitoneal injection of 20 mg/kg PQ). The signal was monitored at 257 nm, the retention time for PQ was ~4.7 min. (**B**) Calibration curve for PQ determination, constructed using PQ standard solutions in blank plasma. (**C**,**D**) Levels of PQ in plasma of males (**C**) and females (**D**) were determined from individual mice as described in *Material and Methods*. Data represent means ± S.D. (*n* = 3–4). A significant genotype- and sex-related difference was not found (*p* > 0.05) at any examined time point.

PQ levels were measured in the plasma of lung-*Cpr*-null and WT control littermates, males and females, at various times following PQ injection. Representative chromatograms and the calibration curve for PQ detection are depicted in Figure 2A,B. The plasma concentration-time curves after a single intraperitoneal PQ administration were similar between males and females and between lung-*Cpr*-null and WT littermates (Figure 2C,D). Calculated pharmacokinetic parameters are shown in Table 1. Small, but statistically significant, sex differences were observed for half-life (*t*_1/2_) in lung-*Cpr*-null mice and for the area under the curve (AUC) and clearance rate (CL/F) in WT mice. However, a difference was not found between lung-*Cpr*-null and WT mice within each sex, for any of the pharmacokinetic parameters determined. These results confirm that the loss of POR expression in the airway epithelial cells did not significantly impact the bioavailability of intraperitoneally injected PQ.

### 3.2. Histological Examination of PQ-Induced Lung Fibrosis in WT and Lung-Cpr-Null Mice

**Figure 3:**
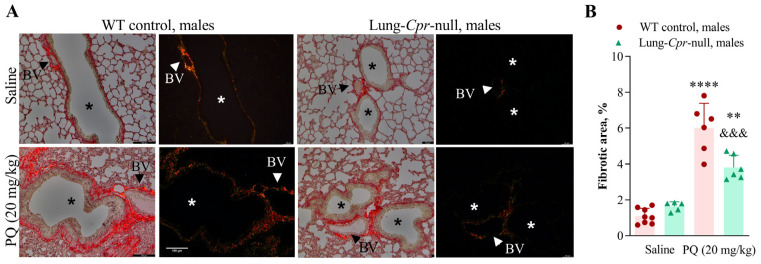
PQ-induced collagen deposition in the lungs of male mice. Three- to four-month old male lung-*Cpr*-null mice and WT control littermates received a single intraperitoneal injection of saline or PQ (20 mg/kg). The lungs were harvested on day 15 after the injection, embedded in paraffin and stained with picrosirius red. The deposition of collagen fibers was evaluated in seven to 12 microscopic fields per lung section, at ×200 magnification, using brightfield (**left**) and polarized light (**right**) microscopy. (**A**) Representative images of lung sections with deposited collagen are shown (BV, blood vessel; *, airway; scale bar is 100 µm). (**B**) The fibrotic lesion area (%) with excessive deposition of collagen was quantified as described in *Material and Methods*. Results represent means ± S.D. (*n* = 5–8 per group). ^&&&^, *p* < 0.001, by mouse genotype; **, *p* < 0.01, ****, *p* < 0.0001, by exposure (one-way ANOVA followed by Bonferroni’s test for multiple comparisons).

**Figure 4:**
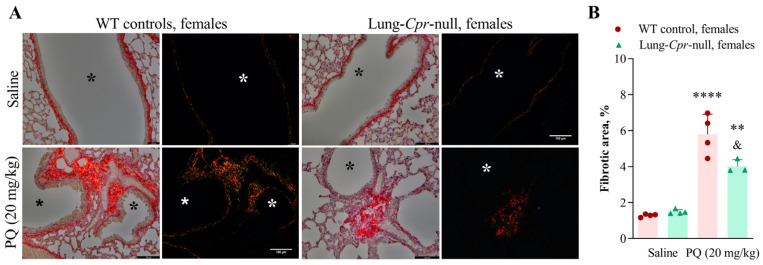
PQ-induced collagen deposition in the lungs of female mice. Three- to four-month-old female lung-*Cpr*-null and WT control littermates received a single intraperitoneal injection of saline or 20 mg/kg PQ. The lungs were collected on day 15 after the injection, embedded in paraffin and stained with picrosirius red. The deposition of collagen fibers was evaluated in seven to eight microscopic fields per lung section, at ×200 magnification, using brightfield (**left**) and polarized light (**right**) microscopy. (**A**) Representative images of lung sections with deposited collagen are shown (*, airway; scale bar is 100 µm). (**B**) The fibrotic lesion area (%) was quantified as described in *Material and Methods*. Results represent means ± SD (*n* = 3–4 per group). ^&^, *p* < 0.05, by mouse genotype; **, *p* < 0.01, ****, *p* < 0.0001, by exposure (one-way ANOVA followed by Bonferroni’s test for multiple comparisons).

Collagen deposition was detected by histological examination of picrosirius red-stained lung sections on day 15 after a PQ injection at 20 mg/kg. Collagen fibers, which stained brightly red when viewed under brightfield, are visible around blood vessels and in the sub-epithelial interstitium in the airways of both control and PQ-treated mice. By viewing sections under polarized light (Figure 3 and Figure 4), we were able to specifically visualize and quantify the birefringence intensity (degree of change in refractive index) of picrosirius red-bound collagen fibers. Images of an entire lung section (7–12 total) were captured and analyzed using Image J. For males, the picrosirius red-stained area was increased following PQ treatment by 5.5-fold (*p* < 0.0001) in the WT group, and by 2.3-fold (*p* < 0.01) in the lung-*Cpr*-null group, compared to genotype-matched saline controls (Figure 3A,B). For females, the PQ-induced increase in picrosirius red-stained area was 4.5-fold (*p* < 0.001) in the WT group and 2.7-fold (*p* < 0.01) in the lung-*Cpr*-null group (Figure 4A,B). In either males or females, the picrosirius red-stained area in PQ-treated mice was significantly lower in the lung-*Cpr*-null group than in the corresponding WT group, by 36.7% (*p* < 0.001) in males and 31.7% (*p* < 0.05) in females. There was no significant sex difference in picrosirius red-stained areas across the genotype and treatment groups. There was a trend of higher deposition of collagen in saline-treated lung-*Cpr*-null males and females than in the sex-matched, saline-treated WT controls, but that was not statistically significant.

### 3.3. Biochemical Analysis of Collagen Levels in Lung Homogenate of WT and Lung-Cpr-Null Mice

**Figure 5:**
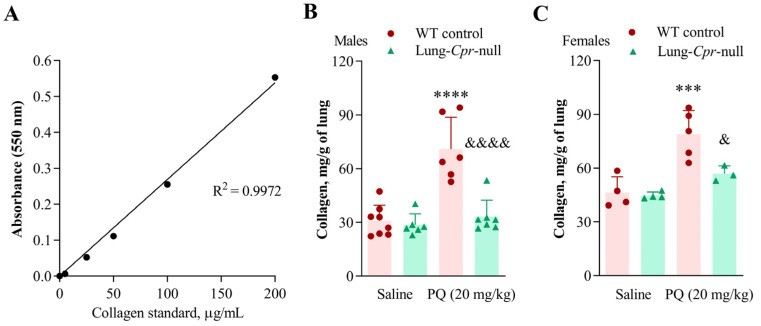
PQ-induced increase in collagen content in mouse lung homogenate. Three- to four-month-old lung-*Cpr*-null mice and WT control littermates received a single intraperitoneal injection of saline or PQ (20 mg/kg). The lungs were harvested for analysis on day 15 after the injections. (**A**) Collagen standard curve for the Sirius red colorimetric plate assay. Signal intensity was linear over the assay range. Each point is the mean of duplicate assays. (**B**,**C**) Sirius-red collagen quantification was performed in lung homogenate for males (**B**) and females (**C**) as outlined in *Material and Methods*. Results represent means ± S.D. (*n* = 3–8 per group). ^&^, *p* < 0.05, ^&&&&^, *p* < 0.0001, by mouse genotype; ***, *p* < 0.001, ****, *p* < 0.0001, by exposure (one-way ANOVA followed by Bonferroni’s test for multiple comparisons).

Biochemical assays were performed to provide a more quantitative assessment of collagen levels in the lung, as a complement to the semi-quantitative image analysis of selected lung sections. Lung homogenate collagen levels, determined using authentic collagen as a standard in a picrosirius red colorimetric plate assay (Figure 5A), were significantly higher in PQ-treated, compared to saline-treated, WT mice, both male (Figure 5B) and female (Figure 5C), but not in lung-*Cpr*-null mice. A single administration of PQ at 20 mg/kg, compared to saline, resulted in 2.3-fold increase (*p* < 0.0001) of total collagen in the lungs of WT controls, but only a 16% increase (*p* > 0.05) in lung-*Cpr*-null mice (Figure 5B), on day 15 after the injection. The collagen content was 47% (*p* < 0.0001) lower in PQ-treated lung-*Cpr*-null males that in PQ-treated WT males. For females, the PQ-induced increase in collagen content in the lungs was 1.7-fold (*p* < 0.001) in WT mice, but only 27% (*p* > 0.05) in lung-*Cpr*-null mice (Figure 5C). The collagen content was 28% (*p* < 0.05) lower in PQ-treated lung-*Cpr*-null females that in PQ-treated WT females. Females showed a trend towards slightly higher content of collagen compared to genotype- and treatment-matched males (Figure 5B vs. Figure 5C). The absolute or relative lung wet weight on day 15 after PQ treatment was similar among all groups (Supplemental Figure S1A–D), which is important as the relative collagen levels were normalized by lung tissue weight. There were no treatment- or genotype-related changes in body weight in either males or females (Supplemental Figure S2A,B).

## 4. Discussion

The purpose of this study was to investigate the role of lung POR in the induction of fibrotic lesions in the lung by PQ in vivo. The unique lung-*Cpr*-null mouse model, with a deletion of the *Por* gene in airway Club cells and AT2 epithelial cells [19,20], was ideally suited for this purpose, as these cells are known targets for PQ-induced redox cycling, oxidative injury, and mitochondrial toxicity in vitro [7,9,10,11,12]. The effectiveness and site specificity of *Por* gene deletion in this mouse model, and confirmation of loss of POR protein expression and function, have been demonstrated previously [19,20], though this is the first time that this mouse model has been utilized to study the direct involvement of POR (instead of the microsomal CYP enzymes that are supported by POR) in xenobiotic toxicity. Our histological and biochemical findings showed that, upon treatment with PQ under conditions that induce lung fibrosis in WT mice, lung-*Cpr*-null mice had significantly reduced collagen deposition in the lungs, in both males and females. This strongly supports the hypothesis that metabolism of PQ by POR in target cells in the lungs is an important contributor to PQ’s lung toxicity. Notably, the finding that WT and null mice had similar plasma PQ levels and pharmacokinetic parameters ensured that the genotype difference in the extent of collagen deposition or collagen content in the lung was not due to a difference in circulating PQ levels. In that connection, the apparent sex difference in systemic PQ metabolism in WT mice (as reflected by lower AUC and higher CL/F in males than in females), which did not lead to a significant difference in collagen deposition or content in the lung, should be interpreted with caution, as the C_max_ value, often a determining factor for acute toxicity, did not have a sex difference (Table 1).

The dose (20 mg/kg) and route (i.p.) of PQ administration, as well as the time point (day 15) for observing PQ toxicity in the lungs, were chosen based on previous publications [29,30]. The ability of PQ to induce pulmonary fibrosis in WT mice has been demonstrated in several other studies, which differed in PQ dosage, post-exposure time and/or route of administration [13,14,15]. Consistent with these previous studies, our histological analysis of lung sections showed that collagen fibers were mostly deposited around blood vessels (in both control and PQ-treated mice) and in the sub-epithelial interstitium in PQ-treated WT mice. The sites of extensive deposition of collagen fibers in PQ-treated mice coincided with the location of collagen-producing cell populations that reside just below the epithelial cells in the conducting airways, surround the bronchovascular bundles, or are embedded in close proximity to the AT2 cells [31].

In lung-*Cpr*-null mice, for both males and females, increased pulmonary accumulation of collagen fibers was also detected in the PQ-treated, compared to saline-treated, groups, but the extent of increase was much lower than in PQ-treated WT animals (Figure 3 and Figure 4). These histological findings were consistent with biochemical measurements of total collagen levels in lung homogenates (Figure 5). Notably, it is reassuring that results from both image analysis and biochemical assays show a significant impact of the POR loss on lung collagen levels. However, an apparent difference was noted between the two sets of data, in that the residual PQ-induced increase in collagen levels in the lung-*Cpr*-null mice was significant when quantified using image analysis (Figure 3B and Figure 4B), but not when quantified using biochemical assays (Figure 5B,C). The discrepancy may be explained by the fact that the biochemical approach, which measures collagen levels in all lung structures (as tissue homogenate), is less sensitive than image analysis for detection of small increases in collagen deposition. It is also possible, though less likely, that lobular difference in the extent of fibrosis might have contributed to the subtle difference in the two data sets, as only one half of the lung was used for histology (left lung) or biochemical analysis (right lung). Overall, these results suggest that, while the POR of airway epithelial cells clearly plays a role in PQ-induced fibroproliferative response in the lungs, it may not be the only enzyme mediator of PQ’s lung toxicity. It should be noted, however, that the residual toxicity seen in the lung-*Cpr*-null mice may or may not be fully attributable to other NADPH-dependent oxidoreductases identified in previous in vitro experiments [16,17,18]. We have shown previously that *Por* deletion did not occur in 18–25% of airway epithelial cells in the lung-*Cpr*-null mice [20]. These residual POR-positive cells may partly account for the residual PQ toxicity in the lung-*Cpr*-null mice. Additionally, POR expressed in other lung cells (e.g., endothelial cells or immune cells) or in other organs, such as the liver, which is intact in the lung-*Cpr*-null mice, would metabolize PQ, generating PQ metabolites that might reach target lung cells to cause toxicity. Thus, further studies that directly modulate activity or expression of these other, non-POR, oxidoreductases are needed to directly assess their possible contribution to PQ toxicity in vivo.

The relative contributions of Club cells and AT2 cells to PQ-induced collagen deposition were not examined in this study. Importantly, the abundance of POR expression in airway epithelial cells (mostly Club cells) is much greater than that in AT2 cells; as we have shown previously, POR was barely detected in AT2 cells using immunohistochemistry, but was readily detected in nearly all airway epithelial cells [19,20]. The efficiency of *Por* deletion in the two types of cells also seemed to differ; while 62–85% of Club cells lose POR expression following induction of Cre expression [20], only ~32% of AT2 cells showed *Por* deletion in an enriched AT2 cell population in this mouse model [19]. Taken together, these data suggest that airway epithelial Club cell POR plays a more important role than AT2 cell POR in mediating PQ’s fibrotic effects in the lung.

The ability of POR and other NADPH-dependent oxidoreductases to mediate PQ redox cycling has been well established [16,17,18]. It is thus reasonable to speculate that, mechanistically, the decrease in PQ-induced lung fibrosis in the lung-*Cpr*-null mice was due to a lowered ability to mediate PQ-induced redox cycling, and the resulting decreases in oxidative damage and subsequent proliferation of fibroblasts in nearby regions as an attempt to regenerate and restore normal architecture of the damaged tissue. However, given the broad involvement of POR-dependent enzymes (all microsomal CYPs and heme oxygenase) in the metabolism of various endogenous compounds [32], it remains to be determined whether other mechanisms also contribute.

PQ induces toxicity in several organs besides the lung, such as the brain, heart, kidney, liver, and skin [33,34,35,36,37,38,39,40]. Toxicity in these other organs was not examined here, given our focus on the lung fibrosis model. However, our findings on the role of lung POR in PQ-induced lung fibrosis suggest that POR may also contribute to PQ’s toxicity in these other organs in vivo. In that connection, other organ-specific *Por*-null mouse models exist, such as liver-specific *Cpr*-null [41] and brain neuron-specific *Cpr*-null [42], or can be prepared through intercrossing between the *Cpr*-lox models [43] and various tissue specific *Cre*-transgenic mouse models, and they can be utilized to address the specific role of target tissue POR in PQ’s toxicity in various affected organs. Notably, PQ has been reported to concentrate in the brain of C57BL/6 mice following systemic administration, which results in a relatively long half-life (~1 month) in this tissue even after a single intraperitoneal injection of 10 mg/kg [44,45,46]. It would be interesting to determine the role of brain POR in PQ bioactivation and toxicity in the brain in a chronic setting.

Human exposure to PQ occurs through multiple routes, including ingestion, inhalation and dermal absorption [1,7]. The present study, utilizing a previously established experimental exposure model [29,30], provides proof-of-concept data on the role of lung POR in bioactivating PQ derived from systemic circulation, though additional bioactivation through other enzymes or organs cannot be excluded. Our findings apply to scenarios of PQ exposure through non-inhalation routes, such as dermal and oral, where the lung is also exposed to PQ through systemic circulation. The results should also apply, at least partly, to scenarios of PQ exposure through inhalation, in which case the lung is exposed to PQ through both direct absorption from air and to PQ that has already entered systemic circulation. However, the relative contributions of different oxidoreductases in lung epithelial cells to PQ bioactivation may differ if the different routes of exposure result in differences in intracellular PQ concentrations. At any rate, the confirmation that systemic levels of PQ were not different between WT and lung-*Cpr*-null mice (Figure 2) indicated similar exposure of the lungs of these mice to PQ, and validated that the differences in extent of fibrosis were not due to a pharmacokinetic change that might have resulted from the genetic manipulations in the mouse model.

## 5. Conclusions

The results of this study show that POR of lung airway epithelial cells contributes to PQ-induced lung fibrosis in the lungs of male and female mice. This is the first study that evaluated and confirmed the important role of POR in PQ-induced pulmonary toxicity in vivo. This finding supports the utility of POR as a molecular target for development of a topically applied antidote for PQ toxicity in the lung.

## Figures and Tables

**Table 1:** Pharmacokinetic parameters for PQ in plasma of lung-*Cpr*-null mice and their control littermates. Plasma levels of PQ from Figure 2C,D were used to calculate the pharmacokinetic parameters, including C_max_ (maximal concentration), *t*_1/2_ (half-life), AUC (area under concentration-time curve), and CL/F (clearance). Values shown are means ± S.D. (*n* = 4 for males and *n* = 3 for females). ^&^, *p* < 0.05, compared to corresponding female value (one-way ANOVA followed by Bonferroni multiple comparisons test).

Mouse Genotype	Sex	C_max_, µg/mL	*t*_1/2_, h	AUC, µg/mL × h	CL/F, L/kg/h
Lung-*Cpr*-null	Male	2.42 ± 0.30	0.83 ± 0.05 ^&^	1.38 ± 0.21	14.8 ± 2.1
	Female	2.41 ± 0.52	0.59 ± 0.10	1.71 ± 0.34	12.0 ± 2.2
WT (littermates)	Male	2.32 ± 0.48	0.85 ± 0.07	1.20 ± 0.25 ^&^	17.1 ± 3.4 ^&^
	Female	2.15 ± 0.27	0.72 ± 0.12	1.88 ± 0.29	10.8 ± 1.9

## Data Availability

The data is contained within the article and supplementary material.

## Supplementary Materials

The following is available online at https://www.mdpi.com/article/10.3390/antiox11020219/s1, Figure S1: Lung weights for mice challenged with saline or 20 mg/kg PQ; Figure S2: Body weights for mice challenged with saline or 20 mg/kg PQ.
