# Supplementary material for: Role of Lung P450 Oxidoreductase in Paraquat-Induced Collagen Deposition in the Lung
Source: Antioxidants (Basel). 2022 Jan 24;11(2):219. doi: 10.3390/antiox11020219 (PMC8868258; doi:10.3390/antiox11020219)
Supplement: Supplementary file 1 [file antioxidants-11-00219-s001.zip › antioxidants-1488829-supplementary.pdf]

Supplemental Data

Role of lung P450 oxidoreductase in paraquat-induced collagen deposition in the lung

Nataliia Kovalchuk[1], Joseph L. Jilek[1], Laura S. Van Winkle[2], Nathan J. Cherrington[1], Xinxin Ding[1]

[1]Department of Pharmacology and Toxicology, College of Pharmacy, University of Arizona, Tucson, AZ, USA

[2] Department of Anatomy, Physiology and Cell Biology, Center for Comparative Respiratory Biology and Medicine, School of Veterinary Medicine and Center for Health and the Environment, University of California at Davis, Davis, CA 95616, USA

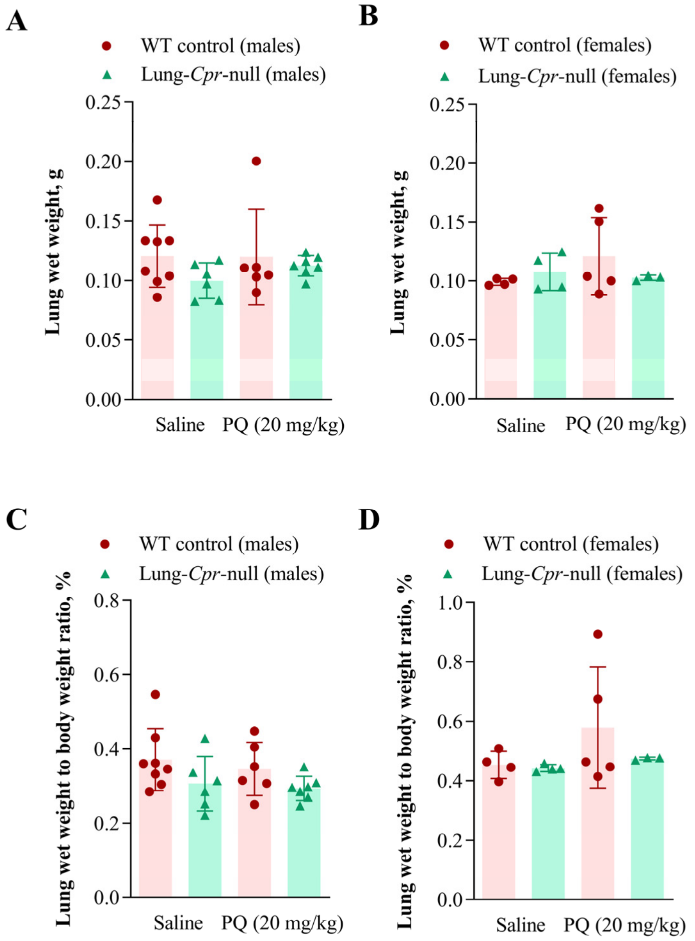

Figure S1. Lung weights for mice challenged with saline or 20 mg/kg PQ. Male (A, C) and female (B, D) lung-*Cpr*-null mice and their WT control littermates received a single intraperitoneal injection of 20 mg/kg PQ in saline or saline alone. The lung weights (A, B) or "lung-weight to body-weight" ratios (C, D) determined on day 15 after the challenge are reported. Values are means ± S.D. (n = 3–8).

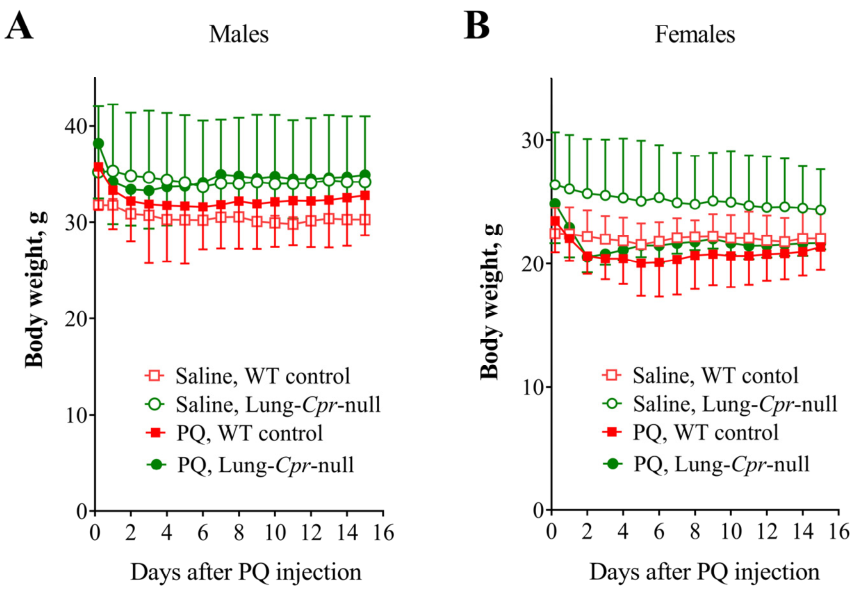

Figure S2. Body weights for mice challenged with saline or 20 mg/kg PQ. Male (A) and female (B) lung-*Cpr*-null mice and their WT control littermates received a single intraperitoneal injection of 20 mg/kg PQ in saline or saline alone. Values are means ± S.D. (*n* = 3–4).
